# Supplementary material for: Assessment of long-term trends in genetic mean and variance after the introduction of genomic selection in layers: a simulation study
Source: Front Genet. 2023 May 10;14:1168212. doi: 10.3389/fgene.2023.1168212 (PMC10206274; doi:10.3389/fgene.2023.1168212)
Supplement: Supplementary file 1 [file DataSheet1.pdf]

Supplementary Material

1 SUPPLEMENTARY TABLES

1.1 Supplementary Table 1

Table S1. Rate of inbreeding (ΔF x100) and effective population size (Ne) based on observed neutral loci heterozygosity (SD x100 over replicates in parentheses).

Breeding program	40 sires per generation			120 sires per generation		
	ΔF/year (x100)	ΔF/generation (x100)	Ne	ΔF/year (x100)	ΔF/generation (x100)	Ne
PTS	5.22 (0.43)	5.22 (0.43)	10	3.11 (0.27)	3.11 (0.27)	16
GTS	4.82 (0.37)	3.62 (0.27)	14	3.58 (0.27)	2.68 (0.20)	19
GTSMF	4.69 (0.23)	3.52 (0.17)	14	3.53 (0.22)	2.64 (0.17)	19
GOCS 45	3.53 (0.28)	2.65 (0.21)	19	2.79 (0.17)	2.09 (0.13)	24
GOCS 65	2.24 (0.20)	1.68 (0.15)	30	1.72 (0.14)	1.29 (0.10)	39

	Fluctuating number of sires per generation		
	ΔF/year (x100)	ΔF/generation (x100)	Ne
UGOCS 45	5.36 (0.45)	4.02 (0.34)	12
UGOCS 55	3.18 (0.14)	2.38 (0.11)	21

PTS - conventional truncation selection; GTS - genomic truncation selection;
GTSMF - GTS with minimization of progeny inbreeding;
GOCS X - genomic optimal contribution selection; UGOCS X - unconstrained GOCS; with the X trigonometric pe

1.2 Supplementary Table 2

Table S2. Rate of inbreeding (ΔF x100) and effective population size (Ne) based on observed QTL heterozygosity (SD x100 over replicates in parentheses).

Breeding program	40 sires per generation			120 sires per generation		
	ΔF/year (x100)	ΔF/generation (x100)	Ne	ΔF/year (x100)	ΔF/generation (x100)	Ne
PTS	5.31 (0.37)	5.31 (0.37)	9	3.17 (0.29)	3.17 (0.29)	16
GTS	4.96 (0.35)	3.72 (0.26)	13	3.72 (0.27)	2.79 (0.20)	18
GTSMF	4.94 (0.25)	3.70 (0.19)	14	3.66 (0.23)	2.74 (0.17)	18
GOCS 45	3.68 (0.31)	2.76 (0.23)	18	2.87 (0.17)	2.16 (0.12)	23
GOCS 65	2.35 (0.22)	1.76 (0.16)	28	1.76 (0.12)	1.32 (0.09)	38

	Fluctuating number of sires per generation		
	ΔF/year (x100)	ΔF/generation (x100)	Ne
UGOCS 45	5.51 (0.44)	4.13 (0.33)	12
UGOCS 55	3.29 (0.14)	2.47 (0.10)	20

PTS - conventional truncation selection; GTS - genomic truncation selection;
GTSMF - GTS with minimization of progeny inbreeding;
GOCS X - genomic optimal contribution selection; UGOCS X - unconstrained GOCS; with the X trigonometric p

1.3 Supplementary Table 3

Table S3. Rate of inbreeding (ΔF x100) and effective population size (Ne) based on pedigree (SD x100 over replicates in parentheses).

Breeding program	40 sires per generation			120 sires per generation		
	ΔF/year (x100)	ΔF/generation (x100)	Ne	ΔF/year (x100)	ΔF/generation (x100)	Ne
PTS	3.36 (0.42)	3.36 (0.42)	17	1.40 (0.20)	1.40 (0.20)	34
GTS	0.89 (0.07)	0.67 (0.06)	75	0.34 (0.02)	0.26 (0.01)	195
GTSMF	0.84 (0.09)	0.64 (0.07)	80	0.34 (0.02)	0.25 (0.02)	196
GOCS 45	0.25 (0.02)	0.19 (0.01)	266	0.11 (0.01)	0.08 (0.01)	616
GOCS 65	0.17 (0.01)	0.13 (0.01)	388	0.06 (0.01)	0.05 (0.01)	1052

	Fluctuating number of sires per generation		
	ΔF/year (x100)	ΔF/generation (x100)	Ne
UGOCS 45	1.12 (0.06)	0.84 (0.05)	60
UGOCS 55	0.26 (0.03)	0.20 (0.03)	254

PTS - conventional truncation selection; GTS - genomic truncation selection;
GTSMF - GTS with minimization of progeny inbreeding;
GOCS X - genomic optimal contribution selection; UGOCS X - unconstrained GOCS; with the X trigonometric pe

2 SUPPLEMENTARY FIGURES

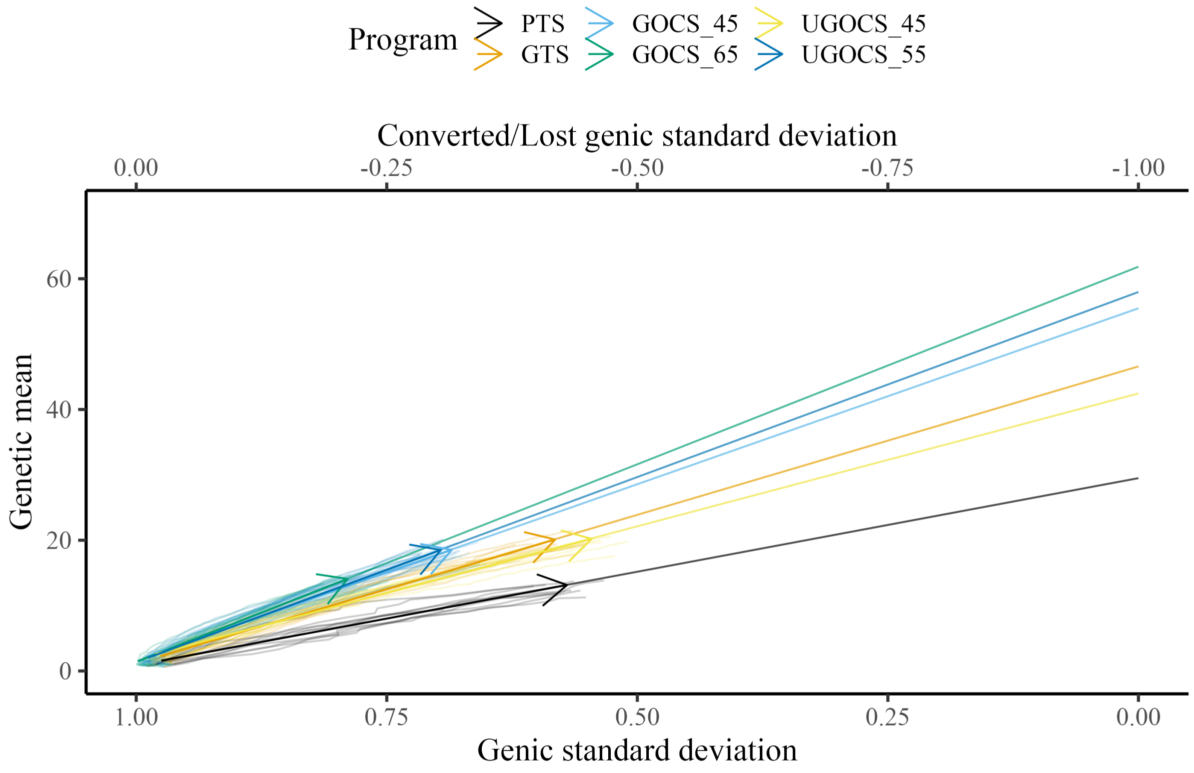

Figure 1a.

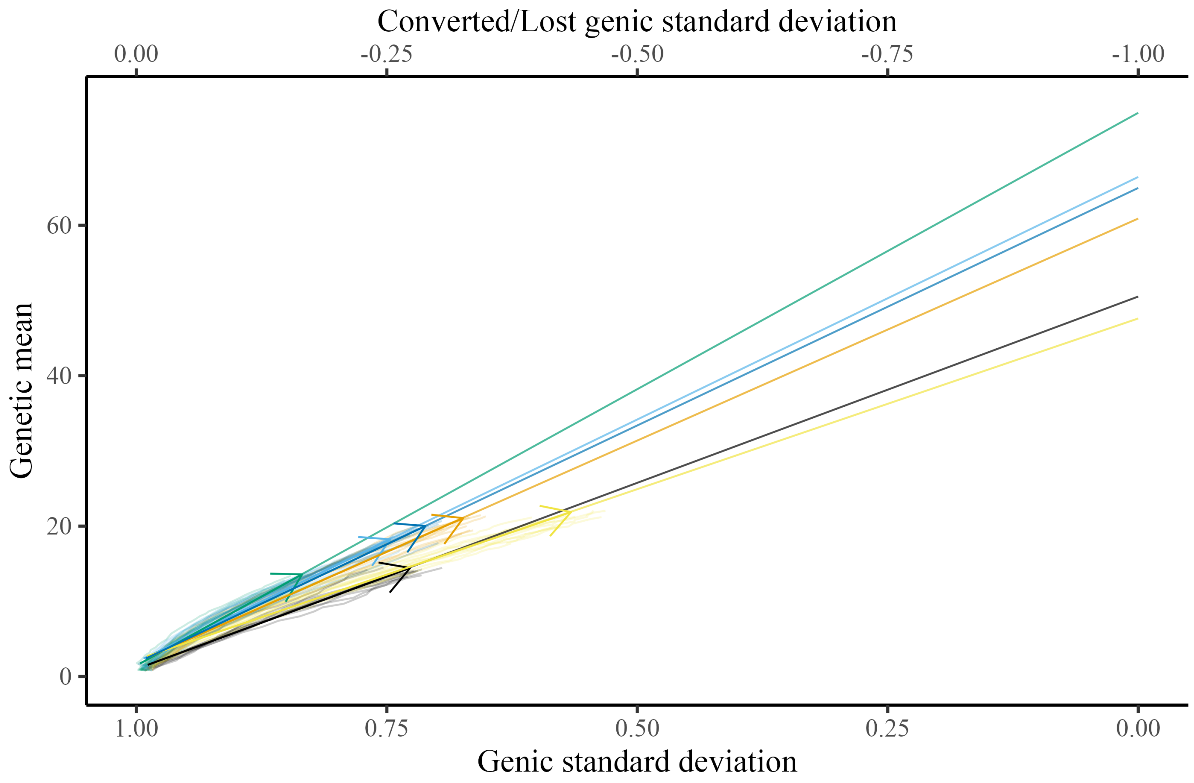

Figure 1b.

Figure 1. Conversion efficiency for conventional truncation selection (PTS) program and genomic programs (genomic truncation selection - GTS, genomic optimal contribution selection - GOCS X, unconstrained GOCS - UGOCS X, with the X trigonometric penalty degrees) marked with an arrow and further extrapolated to 100% of genic variance lost for **(A)** 40 sires and **(B)** 120 sires scenario.

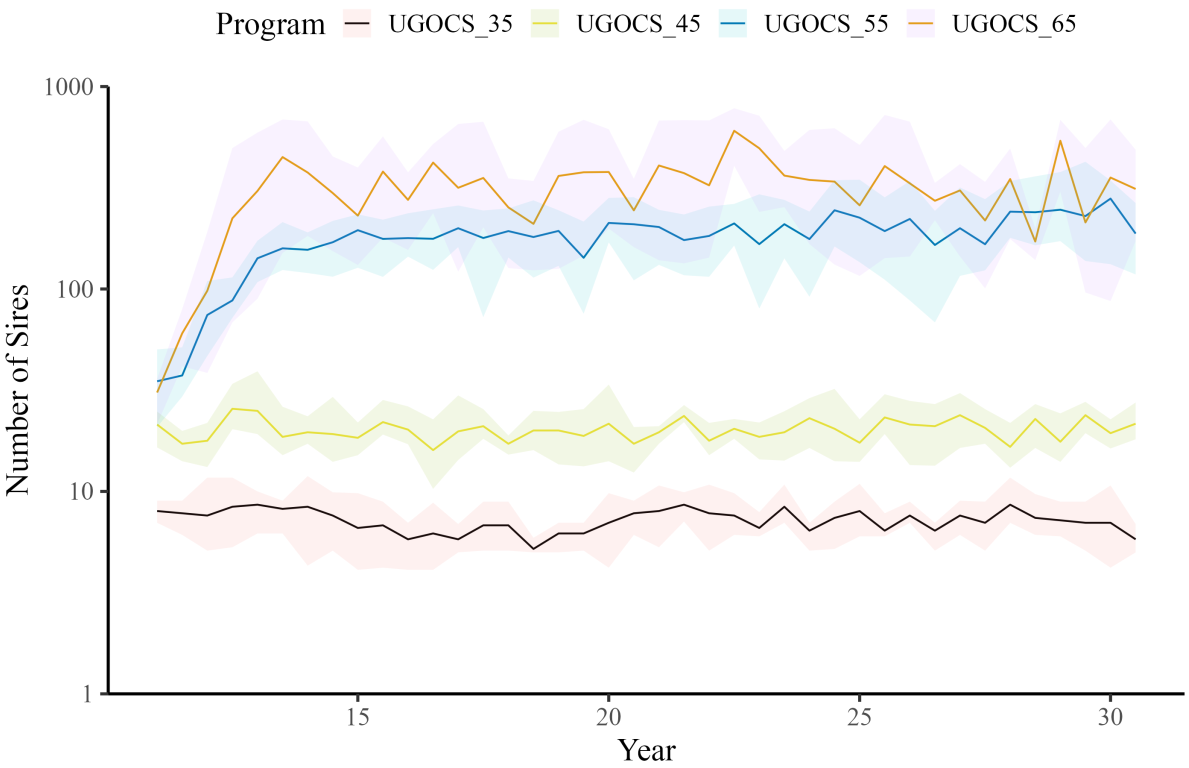

Figure 2a.

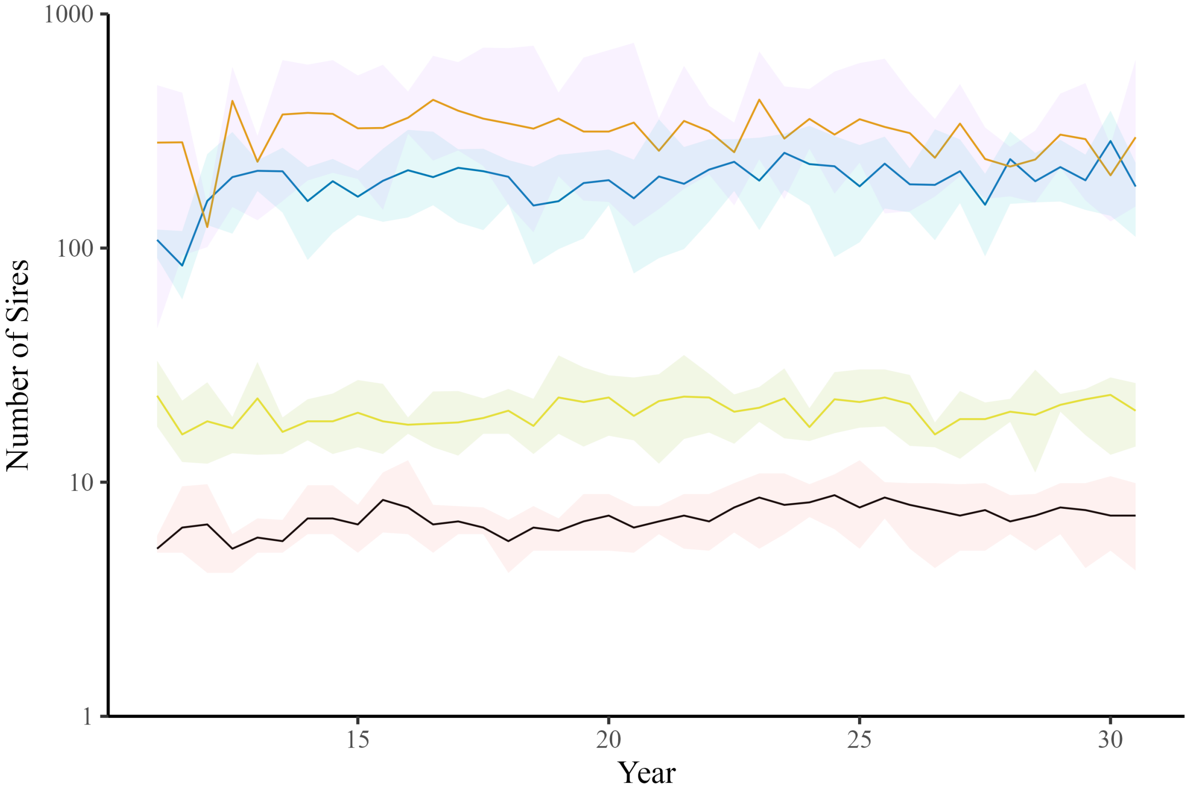

Figure 2b.

Figure 2. Number of sires in each year of genomic unconstrained optimal contribution selection (UGOCS) programs for **(A)** 40 sires and **(B)** 120 sires scenario.

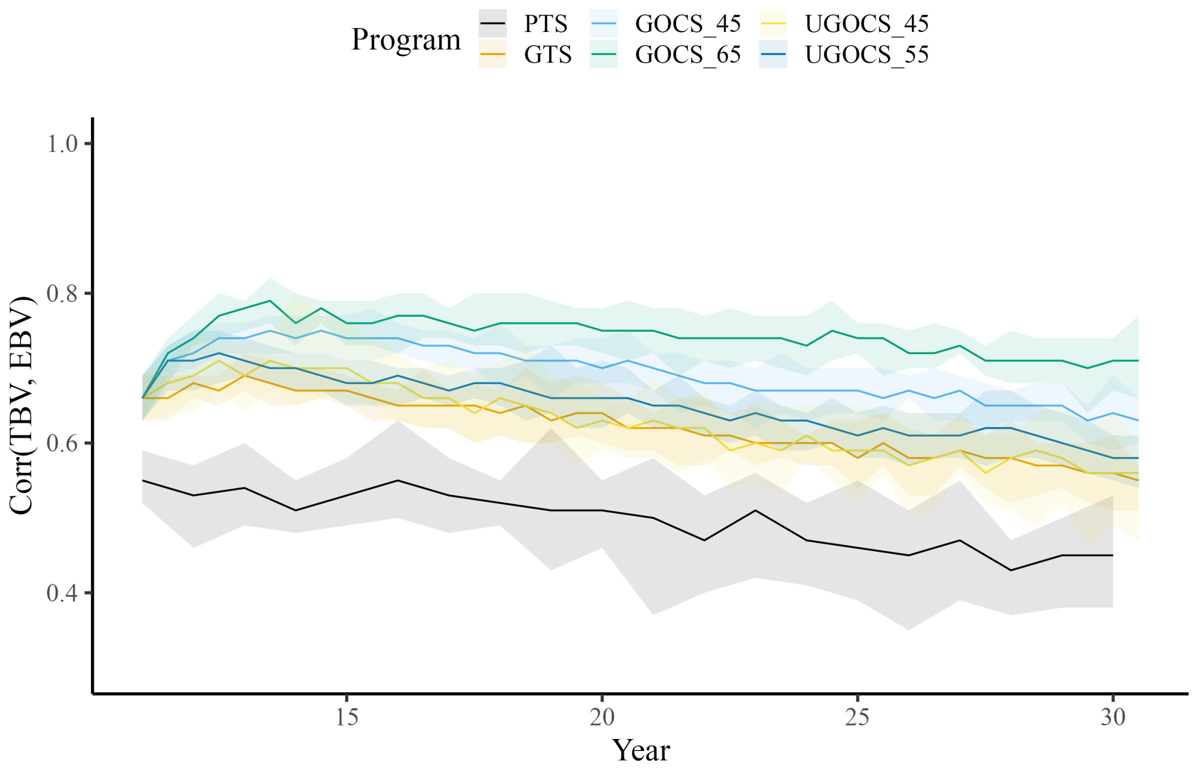

Figure 3a.

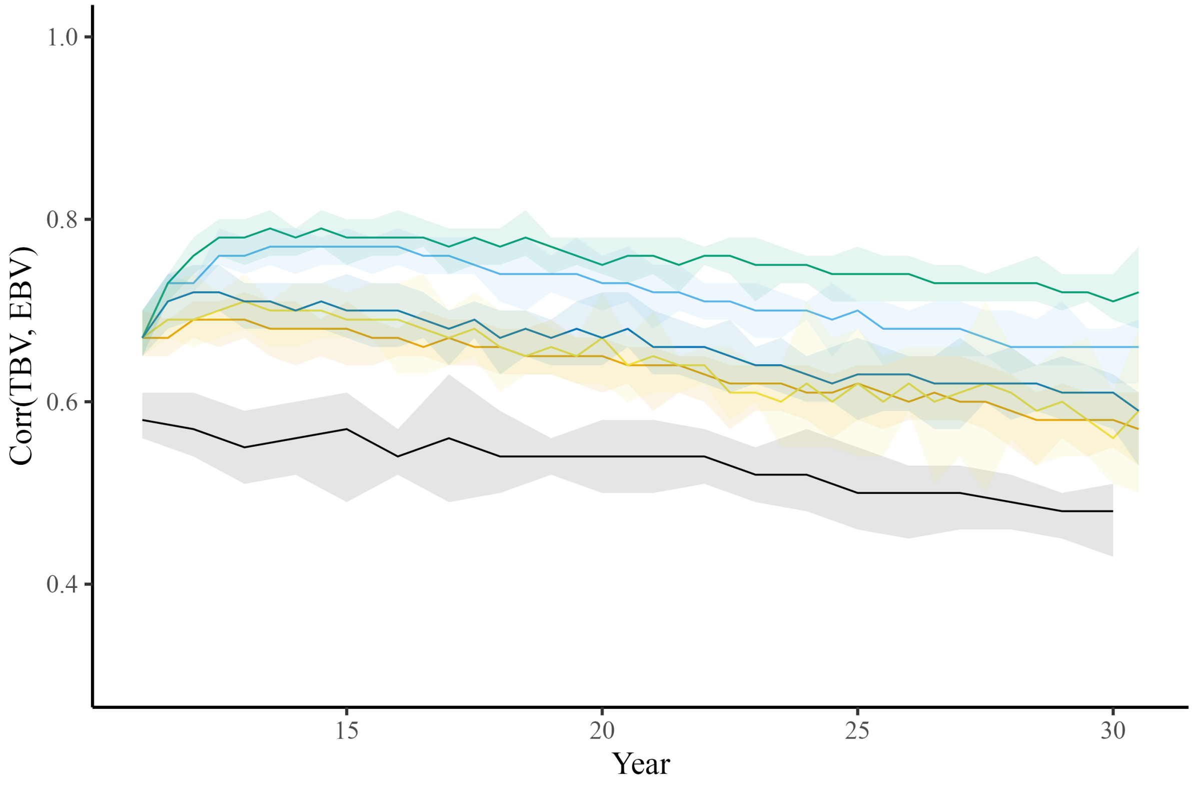

Figure 3b.

Figure 3. Accuracy of selection candidates for conventional truncation selection (PTS) program and genomic programs (genomic truncation selection - GTS, genomic optimal contribution selection - GOCS X, unconstrained GOCS - UGOCS X, with the X trigonometric penalty degrees) across the 20 years of selection for **(A)** 40 sires and **(B)** 120 sires scenario.

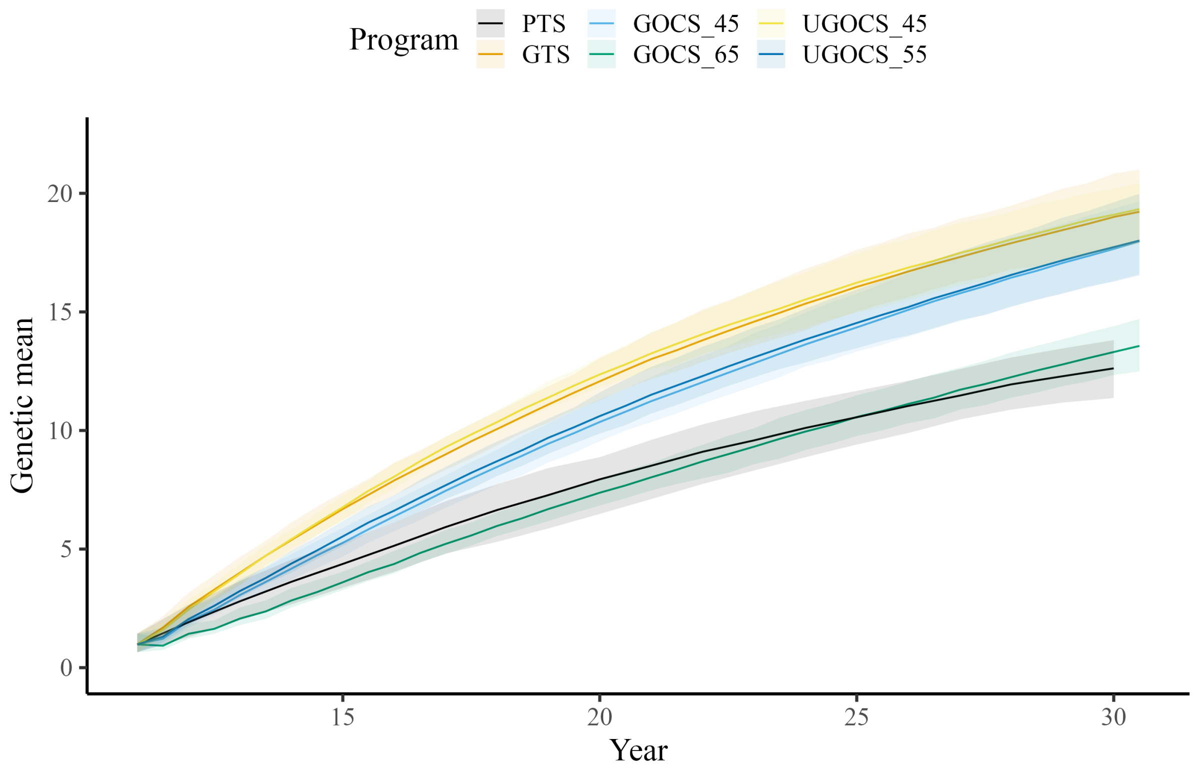

Figure 4a.

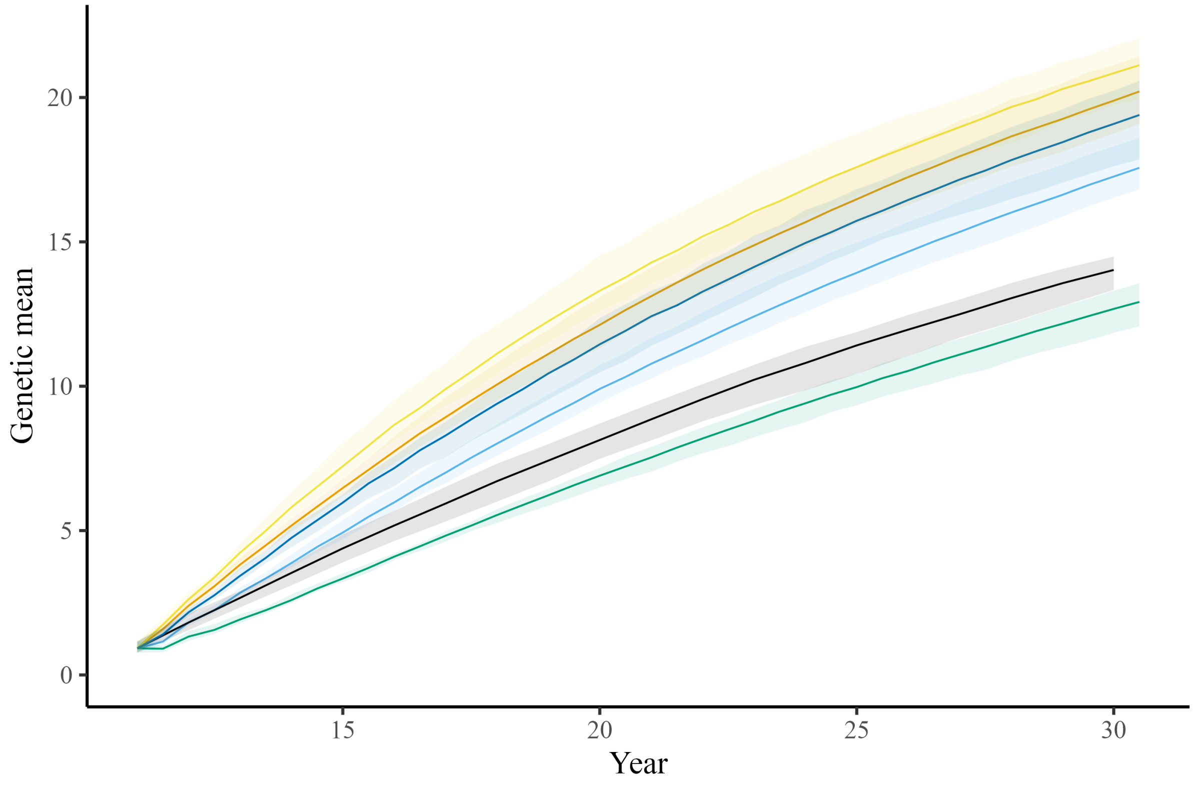

Figure 4b.

Figure 4. Genetic mean of selection candidates for conventional truncation selection (PTS) program and genomic programs (genomic truncation selection - GTS, genomic optimal contribution selection - GOCS X, unconstrained GOCS - UGOCS X, with the X trigonometric penalty degrees) across the 20 years of selection for **(A)** 40 sires and **(B)** 120 sires scenario.

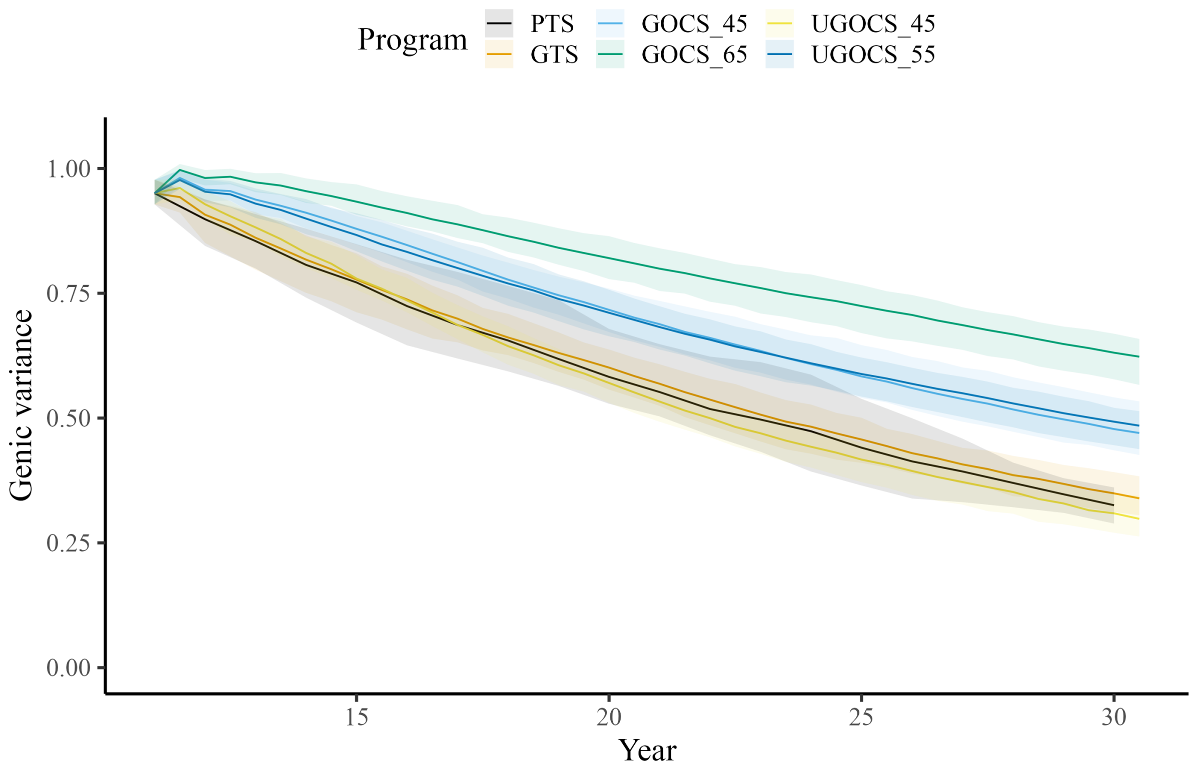

Figure 5a.

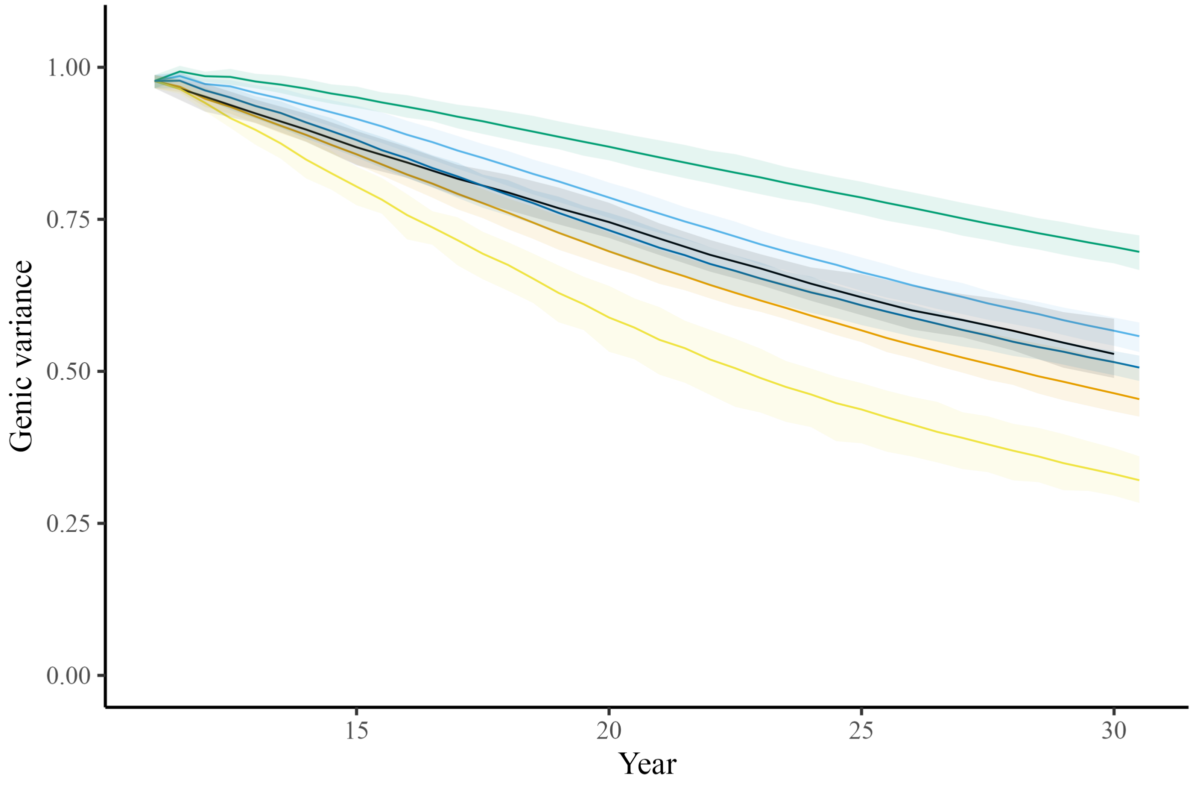

Figure 5b.

Figure 5. Genic variance of selection candidates for conventional truncation selection (PTS) program and genomic programs (genomic truncation selection - GTS, genomic optimal contribution selection - GOCS X, unconstrained GOCS - UGOCS X, with the X trigonometric penalty degrees) across the 20 years of selection for **(A)** 40 sires and **(B)** 120 sires scenario.
